# Supplementary material for: Outcomes and Risk Factors of Critically Ill Patients with Hematological Malignancy. Prospective Single-Centre Observational Study
Source: Medicina (Kaunas). 2021 Nov 30;57(12):1317. doi: 10.3390/medicina57121317 (PMC8707137; doi:10.3390/medicina57121317)
Supplement: Supplementary file 1 [file medicina-57-01317-s001.zip › medicina-1459542_Supplementary.pdf]

Supplementary Materials: The following are available online at www.mdpi.com/xxx/s1,

Supplementary Table S1. Classification of chemotherapy regimens

Non-intensive	Intensive
CyDex	IDA-FLAG
CyBorDex	HDAraC
Venetoclax monotherapy	R-HDArac/TT
R-CVP	Idarubicin and AraC
CTD	7+3
Decitabine	NOPHO ALL-2008
Thalidomide	DHAP-14
Decitabine + Ibrutinib	R-HDMTx
Chlorambucil + Obinutuzumab	BEACOPP
Brentuximab	R-CHOP
Ibrutinib monotherapy	CHOEP
Steroids	MAE
Hydroxycarbamide	GVD
VTD	R-HiCHOP
Arsenic trioxide	R-DHAP
Pomalidomide + Dexamethasone	Idarubicin
Cyclophosphamide	Blinatumomab
R-Chlorambucil	Venetoclax + Decitabine
R-Bendamustine	HDMtx
R-HDMP	TEDDi
Enasidenib	IntReALL 2010
Imatinib	Daratumumab +VTD-PACE
CTD	Active
Gemcitabine	
R-CD	
Azacitidine	
Polatuzumab + Rituximab + Bendamustine	
Bortezomib + Bendamustine	

VTD: bortezomib, thalidomide, dexamethasone; R: rituximab; HDMtx: high dose methotrexate; CVP: cyclophosphamide, vincristine, prednisolone; CyBorDex: cyclophosphamide, bortezomib, dexamethasone; HDMP: high dose methylprednisolone; CD: cyclophosphamide, dexamethasone; TEDDi: temozolomide, etoposide, doxorubicin, dexamethasone, ibrutinib; DHAP: dexamethasone, cisplatin, cytarabine; MAE: mitoxantrone, cytarabine, etoposide; GVD: gemcitabine, vinorelbine, doxorubicin; CHOEP: cyclophosphamide, doxorubicin, vincristine, etoposide, prednisolone; HiCHOP high-dose cyclophosphamide, doxorubicin, vincristine, prednisolone;, CHOP: cyclophosphamide, doxorubicin, vincristine, prednisolone; VTD-PACE: bortezomib, thalidomide, dexamethasone, cisplatin, doxorubicin, cyclophosphamide, etoposide; ACTIVE: actinomycin D, venetoclax, cytarabine; HDAraC: high-dose cytarabine; TT: thiotepa; BEACOPP: bleomycin, etoposide, doxorubicin, cyclophosphamide, vincristine, procarbazine, prednisolone; Ida-FLAG: idarubicin, fludarabine, cytarabine, G-CSF: 7+3: cytarabine, daunorubicin; R-HDMTx: rituximab, high dose methotrexate; R-CHOP: rituximab, cyclophosphamide, doxorubicin, vincristine, prednisolone; R-HiCHOP: rituximab + high dose cyclophosphamide, doxorubicin, vincristine, prednisolone; R-DHAP: rituximab, dexamethasone, cisplatin, cytarabine;

Supplementary Table S2. Plan for laboratory tests

Analyte	Time										
	On arrival at the ICU (Day 1)	Day 2	Day 3	Day 4	Day 5	Day 7	Day 14	Day 28	Day 35	Day 42	Day 49
FBC	✓	✓	✓	✓	✓	✓	✓	✓	✓	✓	✓
CRP	✓	✓	✓	✓	✓	✓	✓	✓	✓	✓	✓
PCT	✓	✓	✓			✓	✓	✓	✓	✓	✓
APTT											
Prothrombin	✓					✓	✓	✓	✓	✓	✓
Fibrinogen	✓					✓	✓	✓	✓	✓	✓
Albumin	✓					✓	✓	✓	✓	✓	✓
Triglycerides	✓					✓	✓	✓	✓	✓	✓
Urea	✓										
Creatinine	✓	✓	✓	✓	✓	✓	✓	✓	✓	✓	✓
Bilirubin	✓	✓	✓	✓	✓	✓	✓	✓	✓	✓	✓
ALAT	✓	✓	✓	✓	✓	✓	✓	✓	✓	✓	✓
ASAT	✓	✓	✓	✓	✓	✓	✓	✓	✓	✓	✓
AGB	✓	✓	✓	✓	✓	✓	✓	✓	✓	✓	✓
Urine analysis	✓					✓	✓	✓	✓	✓	✓
Troponin I	✓					✓	✓	✓	✓	✓	✓
BNP	✓					✓	✓	✓	✓	✓	✓

ICU: intensive care unit; FBC: full blood count; CRP: C-reactive protein; PCT: procalcitonin; APTT: activated partial thromboplastin time; ALAT: alanine aminotransaminase; ASAT: aspartate aminotransferase; ABG: arterial blood gas; BNP: brain natriuretic peptide;

Supplementary Table S3. SOFA scores in ICU

SOFA scores in ICU n (%)					
SOFA score	Day 1	Day 2	Day 3	Day 4	Day 5
0-4	31 (27.2)	22 (20.4)	24 (26.4)	19 (24.7)	14 (22.6)
5-9	62 (54.4)	59 (54.6)	43 (47.3)	44 (57.1)	35 (56.5)
10-14	19 (16.7)	24 (22.2)	21 (23.1)	13 (16.9)	13 (21.0)
15-20	2 (1.8)	3 (2.8)	3 (3.3)	1 (1.3)	0 (0)

SOFA: Sequential Organ Failure Assessment; ICU: intensive care unit;

Supplementary Table S4. Changes of SOFA score in ICU

Changes of SOFA score in ICU n (%)			
SOFA score	Between day 1 – 2	Between day 1 - 3	Between day 2 – 3
Decreased	23 (21.3)	26 (28.6)	37 (40.7)
Increased	66 (61.1)	47 (51.6)	27 (29.7)
Equal	19 (17.6)	18 (19.8)	27 (29.7)

SOFA: Sequential Organ Failure Assessment; ICU: intensive care unit;
